# Neuroinflammatory Gene Expression Pattern Is Similar between Allergic Rhinitis and Atopic Dermatitis but Distinct from Atopic Asthma

**DOI:** 10.1155/2020/7196981

**Published:** 2020-06-10

**Authors:** Paulina Sobkowiak, Wojciech Langwiński, Joanna Nowakowska, Irena Wojsyk-Banaszak, Dawid Szczepankiewicz, Dorota Jenerowicz, Eliza Wasilewska, Anna Bręborowicz, Aleksandra Szczepankiewicz

**Affiliations:** ^1^Department of Pediatric Pulmonology, Allergy and Clinical Immunology, Poznań University of Medical Sciences, Poznań, Poland; ^2^Molecular and Cell Biology Unit, Department of Pediatric Pulmonology, Allergy and Clinical Immunology, Poznań University of Medical Sciences, Poznań, Poland; ^3^Animal Physiology, Biochemistry and Biostructure, Poznań University of Life Sciences, Poznań, Poland; ^4^Laboratory of Allergic Diseases, Department of Dermatology, Poznań University of Medical Sciences, Poznań, Poland; ^5^Department of Pulmonology and Allergology, Clinical University Centre in Gdansk, Gdansk, Poland

## Abstract

**Methods:**

In the study, we included 86 children diagnosed with atopic asthma (*n* = 25), allergic rhinitis (*n* = 20), and atopic dermatitis (*n* = 20) and healthy control subjects (*n* = 21) of Caucasian origin from the Polish population. The blood leukocyte expression of 31 genes involved in neuroinflammatory response (neurotrophins, their receptors, neuropeptides, and histamine signaling pathway) was analysed using TaqMan low-density arrays. The relative expression of selected proteins from plasma was done using TaqMan Protein Assays. Statistical analysis was done using Statistica.

**Results:**

Blood expression of 31 genes related to neuroimmune interactions showed significant increase in both allergic diseases, allergic rhinitis and atopic dermatitis, in comparison to the control group. We found 12 genes significantly increased in allergic rhinitis and 9 genes in which the expression was elevated in atopic dermatitis. Moreover, 9 genes with changed expression in atopic dermatitis overlapped with those in allergic rhinitis. Atopic asthma showed 5 genes with altered expression. The peripheral expression of neuroinflammatory genes in the human study was verified in target tissues (nasal epithelium and skin) in a rat model of allergic inflammation.

**Conclusions:**

A common pattern of neuroinflammatory gene expression between allergic rhinitis and atopic dermatitis may reflect similar changes in sensory nerve function during chronic allergic inflammation.

## 1. Introduction

Neuroimmune interactions were first reported a few decades ago in the pathogenesis of allergic diseases. Mediators released during inflammatory response alter the function of sensory and parasympathetic neurons [1]. Previous studies showed that neurons interact with the immune cells (mainly mast cells, dendritic cells, eosinophils, and Th2 cells) and structural cells (airway epithelium, nasal mucosa, and skin keratinocytes), and these interactions undergo altered regulation during chronic inflammation [2]. Upon allergen exposure, immune and structural cells secrete proinflammatory mediators such as histamine, neurotrophins, or cytokines that activate sensory nerves and mediate clinical symptoms of allergies: itch in the skin, sneezing, and upper and lower airway obstruction. Activated nerves secrete neuropeptides such as calcitonin gene-related peptide, substance P, and vasoactive intestinal peptide that influence function of the immune cells and enhance allergic inflammation.

Previous studies in allergic rhinitis (AR) showed that neurotrophins such as nerve growth factor (NGF) are physiologically expressed in the basal area of the nonaffected epithelium, but in allergic rhinitis, it is expressed through the entire thickness of the epithelium leading to increased number of nerve fibers in the atopic mucosa [3]. Upon allergen provocation, neurotrophins (BDNF, NGF) showed enhanced expression in nasal mucosa and nasal lavage that correlated with symptom severity in allergic rhinitis adult patients [4]. Neurotrophins were also elevated in serum of AR patients [5].

Neuroimmune interactions also play a role in the pathogenesis of atopic dermatitis (AD). Previous studies demonstrated increased sensory nerve density that correlated with increased NGF expression in AD lesions [6–8] indicating that neurogenic inflammation underlies AD development (reviewed recently by Siiskonen and Harvima [9]). In AD, classic “histaminergic” itch is mainly mediated histamine receptors (H1 receptors, but also H4 receptors on cutaneous sensory nerves) [10], but it still remains unclear why antihistamine drugs are not effective in treating skin inflammation in AD. Increased serum neurotrophin levels (NGF, BDF) were also observed in AD patients [11].

Apart from the extensive studies of NGF and BDNF in allergic rhinitis and atopic dermatitis, the reports regarding the other genes related to neurogenic inflammation in allergies are limited and focused mainly on adult populations of patients.

In our previous studies regarding the role of neurogenic inflammation in childhood asthma, we found that serum levels of neurotrophins (NGF, NT-3, and NT-4) may be related to atopic asthma severity [12, 13]. Thus, we hypothesized that the expression of neuroinflammatory genes may be altered also in the other allergic diseases, including atopic dermatitis and allergic rhinitis, and that, compared to atopic asthma, the expression pattern may be disease-specific.

## 2. Materials and Methods

In this study, we included children diagnosed with atopic asthma, allergic rhinitis, and atopic dermatitis and healthy control subjects of Caucasian origin from the Polish population. All participants as well as their parents have given written informed consent. The local ethics committee accepted the project. The study was performed in compliance with the Code of Ethics of the World Medical Association (Declaration of Helsinki). Exclusion criteria for all subjects were lack of written consent, comorbid allergic disease, autoimmune diseases, immunodeficiency, neurological diseases, and parasitic infections.

### 2.1. Patient Group

The study cohort (*n* = 86) included asthma (*n* = 25), allergic rhinitis (*n* = 20), atopic dermatitis (*n* = 20), and healthy control subjects (*n* = 21) aged between 6 and 18 years. The patients were recruited from inpatients treated in the Department of Pediatric Pulmonology, Allergy and Clinical Immunology of Poznan University of Medical Sciences and in the Outpatient Allergology Clinic. The control group was recruited from healthy volunteers.

Asthma diagnosis was made according to GINA recommendation, based on clinical asthma symptoms and lung function test [14]. Spirometry was performed on a LungTest 1000 (MES) according to ERS/ATS guidelines [15]. The patients were recruited during stable asthma and received inhaled steroids only. Systemic steroids, antileukotriene agents, and LABA were withdrawn at least 1 month before inclusion in the study. Allergic rhinitis (AR) diagnosis was based on clinical symptoms (itchy nose, sneezing, rhinorrhea, and nasal obstruction), quality of life (QoL), and duration of disease according to Allergic Rhinitis and its Impact on Asthma (ARIA) criteria [16]. In allergic rhinitis patients, asthma was excluded.

The extrinsic type of atopic dermatitis was diagnosed according to Hanifin and Rajka criteria, based on clinical symptoms, and the severity was assessed using the SCORAD index. At the time of recruitment, the patients were treated with topical steroids/antihistamines only, without systemic immunosuppressive treatment at least 1 month before inclusion in the study.

Atopic background in all patients (asthma, allergic rhinitis, and atopic dermatitis) was confirmed by total IgE level higher than the upper normal limits for age, at least one positive reaction to allergen-specific IgE (d1, d2, gx1, tx1, ex1, and mx2), and positive skin prick test results to at least one common aeroallergen (*D. pteronyssinus*, *D. farinae*, cat, dog, *Alternaria alternata*, *Cladosporium herbarum*, and pollen: grass mix, rye, birch pollen, alder, and hazel) (Allergopharma, Germany).

### 2.2. Control Group

The control group consisted of 21 healthy nonatopic children without symptoms of allergy and asthma in the past and no current symptoms of allergic disease confirmed by clinical examination, normal spirometry results, negative skin prick test results, and normal total IgE level.

### 2.3. Gene Expression Analysis

RNA was isolated from peripheral blood leukocytes with the use of TriPure according to the manufacturer's protocol. Total RNA sample concentration was determined using a NanoDrop 1000 spectrophotometer. The gene expression was analysed using assays spanning the exon-exon junction. The genes analysed in this study included neurotrophins (*BDNF*, *NGF*, *NTF-3*, and *NTF-4*), their receptors (*TrkA*, *TrkB*, *TrkC*, and *TNFRSF1B*), specific kinases (*Fyn*, *PLCɣ*, and *MAP3K1*), neuropeptides (*SP*, *NKA*, and *CGRP*), an enzyme involved in their metabolism (*NEP/CD10*), histamine pathway (*HDC*, *HNMT*, *DAO*, and histamine receptors: H1-H4), neurokines associated with histamine function (*IL-1β*, *IL-6*), H1-PKC*α*-MAPK/MEK1-ERK pathway, ion channel receptors (*TRPA1*, *TRPV1*), and proinflammatory cytokines (*IL-4*, *IL-4R*, *IL-13*, and *TNFα*). For specific amplification and detection of mRNA, we used a TaqMan-based quantitative real-time PCR in a TaqMan low-density array (TLDA) format (Thermo Fisher Scientific) that consisted of 32 TaqMan Gene Expression Assays used for mRNA expression analysis from blood leukocytes on a 7900HT Fast Real-Time PCR System (Thermo Fisher Scientific) according to the manufacturer's protocol. Each sample was done in triplicate. The comparative ddCT method [17] was used for calculating relative quantitation of gene expression after outlier removal and data normalization based on the endogenous control gene expression (18S rRNA) using DataAssist software (Thermo Fisher Scientific). The list of analysed genes and their assay IDs is presented in the supplementary file (table S1).

### 2.4. Protein Quantitation Using TaqMan Protein Assay

Blood samples were taken for tubes with an anticoagulant (EDTA), centrifuged to obtain plasma, and frozen at -80°C for further analysis. Concentration of the total protein amount in plasma was done using a BCA kit (Thermo Fisher Scientific). Selected proteins (neurotrophin-3, neurotrophin-4, and HRH2) were measured in diluted plasma samples using a TaqMan Protein Assay according to the manufacturer's protocol as described previously [18].

The following antibodies were used to design TaqMan Protein Assays: antineurotrophin-3, antineurotrophin-4, and anti-HRH2 (R&D Systems, Minneapolis, USA). Antibodies were labelled with biotin using a Biotin Conjugation Kit (Abcam), and the efficiency of biotinylation was assessed using a Biotin Quantitation Kit (Abcam). Biotinylated antibodies were tested using a forced proximity probe test kit (Applied Biosystems, Foster City, USA). Then, the validated antibodies were labelled using the TaqMan Protein Assay Open Kit (Applied Biosystems, Foster City, USA). The specificity of the protein assays was determined using recombinant proteins as the standard (R&D Systems, Minneapolis, USA). TaqMan Protein Assay (TPA) was performed using plasma samples of allergic rhinitis and atopic dermatitis patients as well as control subjects in diluted plasma (1 : 10) in duplicates. TPA was performed using the TaqMan Protein Assay Core Reagents Kit with MasterMix (Applied Biosystems, Foster City, USA) according to the manufacturer's instructions as described previously [18]. Real-time PCR was performed on an ABI PRISM 7900HT Real-Time PCR System (Applied Biosystems, Foster City, USA). Data were analysed using Protein Assist 1.0 (Applied Biosystems, Foster City, USA) that uses the dCt method to calculate relative protein expression between control samples and allergic samples. Sample dilutions were assayed, and the resulting Ct values were normalized to the sample input, which requires accurate protein quantitation. Each reaction plate included a no-protein control (NPC) to calculate dCt values (Ct‐value [sample]–Ct‐value [NPC]). Then, a linear range was generated for each sample, and a dCt threshold was designated. The fold-change between samples was calculated between the crossover points of each linear trend line at the dCt threshold as described previously [19].

### 2.5. Animal Model of Allergic Inflammation

In the study, we used Brown Norway male rats to model allergic asthma induced by house dust mite inhalation. The study was approved by the local ethical committee (agreement no. 35/2017). Six male Brown Norway rats were purchased from Javier Labs (France) with the baseline weight of 170 g ± 15 g. The animals were housed and kept for one week for acclimatization under standard conditions. Then, the animals were randomly allocated into two experimental groups: allergic (*n* = 3) and control groups (*n* = 3). Rats were sensitized by subcutaneous injection of 250 *μ*l house dust mite (HDM) extract (45 *μ*g, Citeq Biologics) in 4% Al(OH)_3_ (Thermo Fisher Scientific, USA) as an adjuvant. Control rats were injected with an equivalent volume of adjuvant. Injections were done once a week for 3 weeks. After that time, animals from the asthmatic group were receiving intranasally HDM extract (120 *μ*g/50 *μ*l three times per week for 4 weeks). Rats from the control group were intranasally exposed to the same volume of PBS with the same experimental schedule. After that, rats were sacrificed by decapitation, and tissues were immediately collected, snap frozen in liquid nitrogen, and stored at -80°C prior to RNA extraction. Histology of lungs was done with hematoxylin/eosin staining to visualize pathological changes in the sensitized rats. Serum IgE was also evaluated to confirm inflammatory changes in the blood. RNA from the nasal epithelium and skin was extracted and isolated using EXTRAzol (DNA-Gdańsk, Poland) and suspended in RNase-free water. For reverse transcription, we used 100 ng of total RNA using a GoScript Reverse Transcription kit (Promega, Poland), and then cDNA was amplified using a GoTaq qPCR Master Mix (Promega, Poland) and specific primers. For gene expression in tissues, we used *Gapdh* as a reference gene. The primer sequences are shown in supplementary table 2. Amplification was done on ABI PRISM 7900HT (Applied Biosystems, USA). Data were analysed with DataAssist v.301 Software (Thermo Fisher Scientific, USA) using the relative quantification method.

### 2.6. Statistical Analysis

The data were checked for normal distribution using a Shapiro-Wilk test. For comparisons of normally distributed data, we used one-way analysis of variance (ANOVA). For comparisons of data that deviated from normal distribution, nonparametric Mann–Whitney *U*-test was used. Normalized expression data were visualized using the Heatmapper tool [20]. Calculations were performed using the Statistica version 12 software. *p* value lower than 0.05 was considered significant. All significance tests were two-tailed.

## 3. Results

### 3.1. Clinical Study

The study cohort (*n* = 86) consisted of patients with allergic rhinitis, atopic dermatitis, and atopic asthma and healthy children from a Polish population of Caucasian origin (Table 1).

Populations were similar in gender and age between the studied diseases and the control group, despite the atopic dermatitis patients who were significantly younger from the nonatopic control (*p* = 0.015). The allergic patients showed significantly higher IgE levels than the control group and significantly lower parameters in spirometry (for atopic asthma and allergic rhinitis) than the control group. The highest blood eosinophil counts were above normal in all allergic diseases and were the highest in atopic dermatitis patients.

### 3.2. Neuroinflammatory Gene Expression in Human Blood

**Figure 1:**
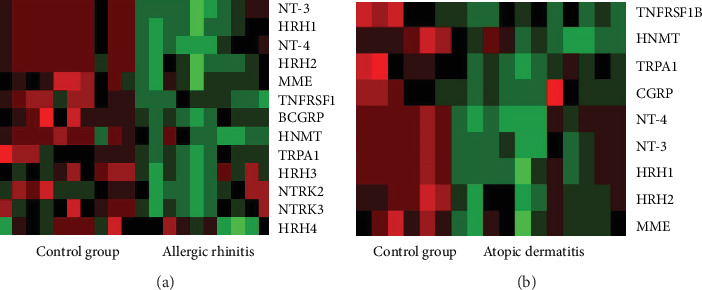
Heat maps for gene expression analysis in allergic rhinitis patients (AR) (reference: control group) (a) and atopic dermatitis (AD) patients (reference: control group) (b).

Analysis of mRNA expression of 31 genes related to neuroimmune interactions showed significant increase in both allergic diseases, allergic rhinitis and atopic dermatitis, in comparison to the control group. We found 12 genes with significantly increased expression in allergic rhinitis and 9 genes in which expression was elevated in atopic dermatitis (Figures 1(a) and 1(b)). Moreover, all the genes with changed expression in atopic dermatitis overlapped with those in allergic rhinitis: *HNMT*, *TRPA1*, *CGRP*, *NT-3*, *NT-4*, *HRH1*, *HRH2*, *MME*, and *TNFRSF1b*.

**Figure 2:**
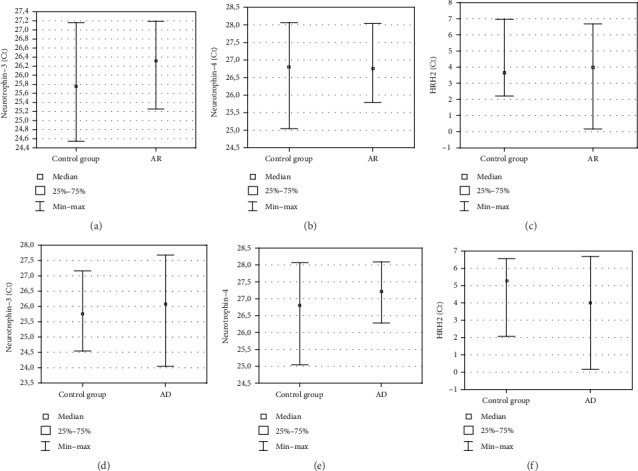
Comparison of plasma levels for neurotrophin-3, neurotrophin-4, and HRH2 proteins in allergic rhinitis (a, b, c, respectively) and atopic dermatitis patients versus control group (d, e, f, respectively) (Mann–Whitney *U*-test).

The highest increase was observed for neurotrophin-3, neurotrophin-4, and HRH2; therefore, they were selected for further verification on the protein level in plasma. Their expression did not differ significantly between the patient groups and the control group (*p* > 0.05) (Figure 2).

**Figure 3:**
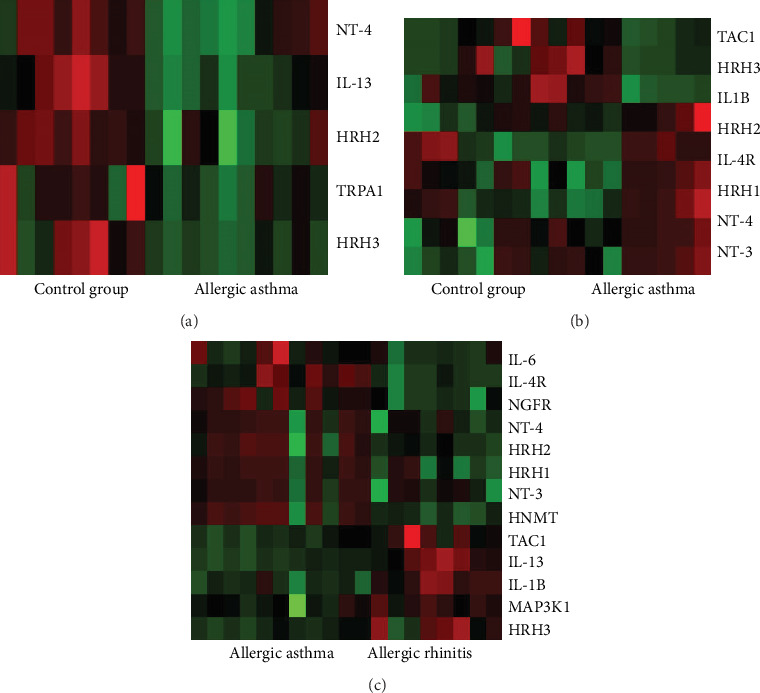
Heat maps for gene expression analysis in allergic rhinitis patients (reference: atopic asthmatic patients) (a), atopic dermatitis patients (reference: atopic asthmatic patients) (b), and atopic asthmatic patients (reference: control group).

In atopic asthma, 5 genes showed significantly different expression as compared to the control group (Figure 3(a)). Three of them were also altered in the other patient groups: *NT-4*, *HRH2*, and *TRPA1*. Interestingly, when we compared the gene expression pattern of allergic rhinitis or atopic dermatitis with atopic asthma, it turned out that, although it was similar between allergic rhinitis and atopic dermatitis, it differed significantly when compared with atopic asthma (Figures 3(b) and 3(c)).

### 3.3. Tissue-Specific Gene Expression in Animal Model of Allergy

**Figure 4:**
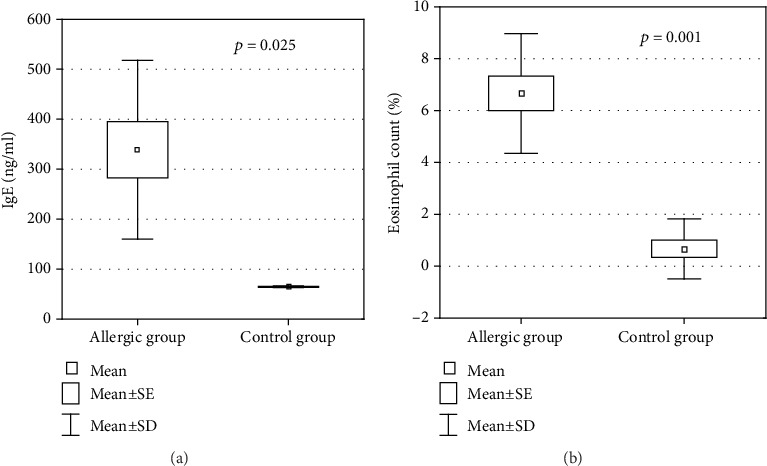
The comparison of total IgE concentration (a) and eosinophil counts (b) between allergic rats and the control group.

**Figure 5:**
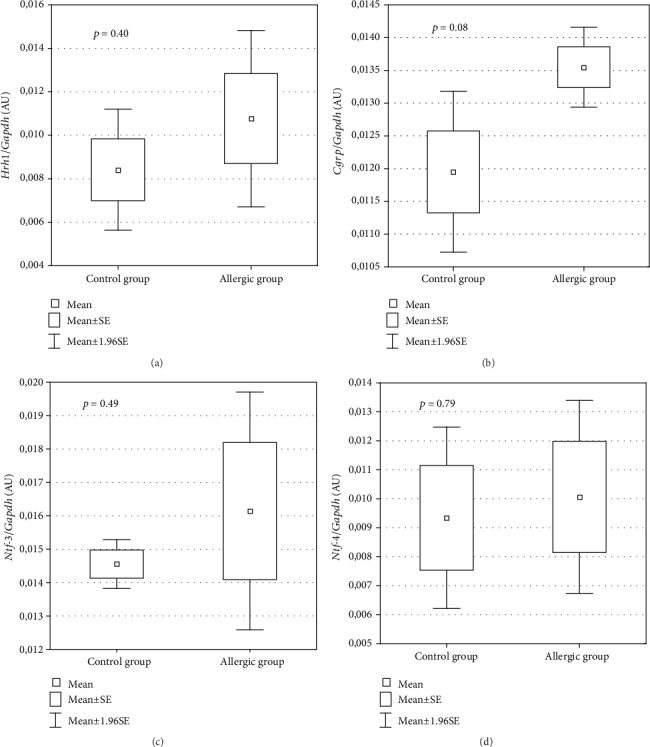
The comparison of tissue-specific expression of *Hrh1* and *Cgrp* in the nasal epithelium (a, b) and *Ntf-3* and *Ntf-4* in the skin (c, d) between allergic and control rats (*t*-test, *n* = 6).

To verify if the altered expression of the 9 genes overlapping between allergic rhinitis and atopic dermatitis in peripheral blood also shows analogous changes in the target tissues (nasal epithelium, skin), we used a rat model of allergic inflammation confirmed by histological analysis and increased the total IgE level and eosinophil counts (Figure 4). The gene expression analysis showed no significant differences in the expression of 9 genes either in the nasal epithelium or in the skin between the sensitized rats and the control group (Figure 5). However, 4 genes showed substantial tissue-specific upregulation in the nasal epithelium (*HRH1*, *CGRP*) or the skin (*NTF-3*, *NTF-4*).

## 4. Discussion

The main observation from this study is that the expression pattern of neuroinflammatory genes in blood leukocytes of allergic children is similar between allergic rhinitis and atopic dermatitis but differs from that observed in atopic asthma.

The common genes that showed significantly altered expression in both allergic rhinitis and atopic dermatitis include 9 genes: *NT-3*, *NT-4*, *HRH2*, *HNMT*, *HRH1*, *TRPA1*, *MME*, *TNFRSF1B*, and *CGRP*. The 3 genes common for all allergic diseases include the upregulation of *NT-4*, *HRH2*, and *TRPA1*. Surprisingly, despite similarities in organ manifestations between allergic rhinitis (upper airways) and atopic asthma (lower airways), we observed that the neuroinflammatory gene expression pattern displayed in allergic rhinitis was more similar to atopic dermatitis than to atopic asthma, suggesting that they share common pathogenesis in regard to neuroimmune interactions. However, the differences between those with allergic diseases and healthy children observed at the gene expression level were not confirmed on the protein level of NT-3, NT-4, and HRH2.

Increased expression of neurotrophin-3 and neurotrophin-4 was previously reported in allergies by several papers, although they were not studied as extensively as NGF or BDNF. In allergic rhinitis, neurotrophin-3 previously showed higher staining scores in nasal mucosa in the adult AR patients than in the control group, whereas its serum level did not differ significantly in AR patients [21]. In atopic dermatitis, increased NT-3 levels were found both locally (mast cells from skin lesions) and in the periphery (plasma) [22]. The authors also found that in a cell culture model of human keratinocytes, increasing concentration of NT-3 suppressed the secretion of IL-8 which may be correlated to functional consequences of AD such as increased susceptibility to microorganisms. In AD skin lesions, increased neurotrophin-4 expression was also reported [23]. These neurotrophins (NT-3 and NT-4) upregulated the chemotaxis of eosinophils from atopic dermatitis adult patients [5] suggesting the role of these neurotrophins in mediating eosinophilic inflammation.

The other genes that showed significantly increased expression in our study were two histamine receptors (*HRH1*, *HRH2*) and the main histamine metabolizing enzyme (*HNMT*). HRH1 is expressed in many tissues including nerves, respiratory epithelium, skin, and immune cells and is activated by histamine that enhances Th1 and Th2 responses leading to hypersensitivity symptoms in the skin and airways [24]. HRH1 function has been extensively studied in the context of AD, as histamine-induced itch is mediated mainly by this receptor [25]. Interestingly, antihistamine drugs targeting this receptor type are often ineffective in AD [26]. However, its blood expression was not previously studied either in AR or in AD in the pediatric population. Another receptor implicated in allergic inflammation is HRH2 that previously showed increased blood expression in adult AR patients during the pollen season [27], which is consistent in our observation in AR pediatric patients. This receptor type was not previously studied in AD, but taking into account inefficient treatment of itch with H1 antihistamines and increased expression of HRH2 in AD children in our study, it may be suggested that histamine type 2 receptors are also involved in mediating allergic itch in children. A previous study showed that one of the major histamine degradation enzymes, histamine-N-methyltransferase (HNMT), was increased in AD patients [28] which corresponds to our findings. In AR, *HNMT* plasma levels were not studied in those patients, either in adults or in children.

Another gene that showed significantly increased expression in both AR and AD is the calcitonin-related polypeptide alpha CGRP. This neuropeptide is released from sensory nerves upon stimulation that acts directly on vascular endothelial cells and smooth muscle cells [29]. The previous study reported that CGRP enhanced antigen presentation and Th2 responses, but inhibited Th1 response [30]. A previous study in an animal model showed the involvement of CGRP in airway inflammation [31], so its upregulation may underlie pathogenesis of allergic diseases.

Neutral endopeptidase (*CD10/NEP*) encodes enzyme that inactivates several peptide hormones including neuropeptides (e.g., substance P or neurotensin). A previous study found that NEP-positive staining was enhanced in the airway epithelium of asthmatic patients treated with inhaled steroids as compared with nonsteroid users. The study suggested that upregulation of NEP mediates the anti-inflammatory effect of steroids in asthmatic airways [32]. Therefore, peripheral upregulation of NEP observed in allergic rhinitis and atopic dermatitis in our study seems to enhance allergic responses.

TNF receptor superfamily member 1B (TNFRSF1B) may nonspecifically bind all neurotrophins and thus modulate the function of specific neurotrophin receptors (NTRK1, 2, and 3). A previous study showed that the number of T cells expressing TNFRSF1B was significantly higher in allergic rhinitis patients [33] and that its expression was increased in nasal biopsies from allergic rhinitis patients as compared to control subjects [34]. Here, we report that its expression is also increased in peripheral leukocytes in allergic patients.

TRPA1 encodes the ion channel and is expressed in sensory neurons that innervate various tissues (including the skin and airway epithelium) [35]. Overexpression of TRPA1 was observed in atopic and allergic contact dermatitis accompanied by chronic skin itching [36, 37] as well as inflammatory processes in the lungs, bronchi, and trachea. Inactivation of the TRPA1 channel decreased the production of cytokines, chemokines, and neurotransmitters and reduced the hyperactivity of respiratory pathways in various animal models [38]. A recent study by Guo et al. [39] showed that upregulation of TRPA1 correlated with chronic cough in an animal model (guinea pigs).

Verification of our findings from a human study in the rat model of allergic inflammation showed that neuroinflammatory genes show increased mRNA expression also in target tissues: *Hrh1* and *Cgrp* in the nasal epithelium and *Ntf-3* and *Ntf-4* in the skin of allergic rats. This observation further suggests the involvement of *HRH1* and *CGRP* in allergic inflammation in the airways as shown previously [31], whereas neurotrophin-3 and neurotrophin-4 are increased in the skin during allergic inflammation which is consistent with previous findings [5, 22, 23].

The main limitation of this study is the relatively small sample size of the analysed groups (approximately 20 subjects in each group); however, these patients underwent careful clinical assessment that enabled us to observe expression patterns specific for allergic diseases. Moreover, the sample size in this study was similar to the previous studies regarding the analysis of neuroinflammatory gene expression.

## 5. Conclusions

In conclusion, our study showed that the neuroinflammatory gene expression pattern is similar between allergic rhinitis and atopic dermatitis patients, suggesting similar changes in sensory nerve function during chronic allergic inflammation. Tissue-specific expression analysis confirmed increased expression of four of these genes in the nasal epithelium and skin in allergic rats.

## Figures and Tables

**Table 1:** Clinical description of the study groups.

	Control group (*n* = 21)	Atopic asthma (*n* = 25)	Allergic rhinitis (*n* = 20)	Atopic dermatitis (*n* = 20)
Gender, male (%)	38	56	55	42
Age, years (mean ± SD)	10.9 ± 2.8	9.31 ± 3.54	11.5 ± 3.8	7.1 ± 4.9^∗^
Positive SPT (%)	0.0	80.0	100.0	90.0
FEV1/FVC pred (mean ± SD)	99.9 ± 5.6	90.6 ± 15.6^∗^	93.3 ± 10.2^∗^	—
FEV1% pred (mean ± SD)	99.9 ± 9.5	83.6 ± 13.5^∗^	96.3 ± 11.1^∗^	—
PEF% pred (mean ± SD)	97.7 ± 12.8	76.6 ± 16.8^∗^	85.8 ± 20.6^∗^	—
exNO (ppb) (mean ± SD)	<20	20.5 ± 15.8	23.2 ± 9.6	—
Blood eosinophils (%) (mean ± SD)	—	5.8 ± 3.4	5.4 ± 3.7	12.8 ± 8.4
IgE (IU/ml) (mean ± SD)	8.4 ± 9.4	257.5 ± 326.7^∗^	77.0 ± 48.8^∗^	1644 ± 3088^∗^

^∗^Indicates significant difference (*p* < 0.05) as compared to the control group.

## Data Availability

The data used to support the findings of this study are available from the corresponding author upon request.

## Supplementary Materials

Supplementary Materials Supplementary materials contain supplementary table 1 that lists all analysed genes and their expression assay IDs and supplementary table 2 that contains primer sequences for gene expression analysis in the rat model of allergic inflammation.
