# Supplementary material for: Neuroinflammatory Gene Expression Pattern Is Similar between Allergic Rhinitis and Atopic Dermatitis but Distinct from Atopic Asthma
Source: Biomed Res Int. 2020 Jun 10;2020:7196981. doi: 10.1155/2020/7196981 (PMC7305544; doi:10.1155/2020/7196981)
Supplement: Supplementary Materials — contain supplementary table 1 that lists all analysed genes and their expression assay IDs and supplementary table 2 that contains primer sequences for gene expression analysis in the rat model of allergic inflammation. [file 7196981.f1.docx]

Supplementary files

Table S1. List of analyzed genes and their assay IDs

| Gene symbol | Function | Assay ID |
| --- | --- | --- |
| *18S rRNA* | Reference gene | 18S-Hs99999901_s1 |
| *ABP1* | amiloride binding protein 1 (amine oxidase) | ABP1-Hs00175631_m1 |
| *BDNF* | brain-derived neurotrophic factor | BDNF-Hs00538277_m1 |
| *CGRP* | calcitonin-related polypeptide alpha | CGRP-Hs01108255_g1 |
| *FYN* | FYN proto-oncogene, Src family tyrosine kinase | FYN-Hs00176628_m1 |
| *HDC* | histidine decarboxylase | HDC-Hs00157914_m1 |
| *HNMT* | histamine N-methyltransferase | HNMT-Hs02759756_s1 |
| *HRH1* | histamine receptor H1 | HRH1-Hs00911670_s1 |
| *HRH2* | histamine receptor H2 | HRH2-Hs00254569_s1 |
| *HRH3* | histamine receptor H3 | HRH3-Hs00200610_m1 |
| *HRH4* | histamine receptor H4 | HRH4-Hs00222094_m1 |
| *IL13* | interleukin 13 | IL13-Hs01124272_g1 |
| *IL1B* | interleukin 1 beta | IL1B-Hs00174097_m1 |
| *IL4* | interleukin 4 | IL4-Hs00174122_m1 |
| *IL4R* | interleukin 4 receptor | IL4R-Hs00166237_m1 |
| *IL6* | interleukin 6 | IL6-Hs00174131_m1 |
| *MAP3K1* | mitogen-activated protein kinase kinase kinase 1 | MAP3K1-Hs00394890_m1 |
| *MAPK1* | mitogen-activated protein kinase 1 | MAPK1-Hs01046830_m1 |
| *MME* | membrane metallo-endopeptidase | MME-Hs00153519_m1 |
| *NGF* | nerve growth factor | NGF-Hs00171458_m1 |
| *TNFRSF1B* | TNF receptor superfamily member 1B | TNFRSF1B -Hs00609976_m1 |
| *NTF3* | neurotrophin 3 | NTF3-Hs00267375_s1 |
| *NTF4* | neurotrophin 4 | NTF4-Hs01921834_s1 |
| *NTRK1* | neurotrophic tyrosine kinase, receptor, type 1 | NTRK1-Hs01021011_m1 |
| *NTRK2* | neurotrophic tyrosine kinase, receptor, type 2 | NTRK2-Hs01093103_m1 |
| *NTRK3* | neurotrophic tyrosine kinase, receptor, type 3 | NTRK3-Hs00176797_m1 |
| *PLCG1* | phospholipase C, gamma 1 | PLCG1-Hs01008225_m1 |
| *PRKCA* | protein kinase C, alpha | PRKCA-Hs00176973_m1 |
| *TAC1* | tachykinin, precursor 1 | TAC1-Hs01105615_m1 |
| *TNFA* | tumor necrosis factor alpha | TNF-Hs00174128_m1 |
| *TRPA1* | transient receptor potential cation channel A1 | TRPA1-Hs00175798_m1 |
| *TRPV1* | transient receptor potential cation channel V1 | TRPV1-Hs00950004_m1 |

Table S2. The sequences of primers for gene expression analysis in the rat model of allergic inflammation.

| Gene | Forward | Reverse |
| --- | --- | --- |
| *Gapdh* | 5’ATCATCAGCAATGCCTCCT3’ | 5’TTCCACGATACCAAAGTTGTC3’ |
| *Hrh1* | 5’TGAGGTACCGAACCAAGACC3’ | 5’ CACTTGTCTTCCCGAAGCTC3’ |
| *Hrh2* | 5’GCAAGCCACAACTCTCACAA3’ | 5’CTTCAAGGGCTTCTCCTCCT3’ |
| *Ntf-3* | 5’GATCCAGGCGGATATCTTGA3’ | 5’AATCATCGGCTGGAATTCTG3’ |
| *Ntf-4* | 5’TCTTGTCTCCCCGAGTGG3’ | 5’GGTGCCGTCTCACTCACC3’ |
| *Cgrp* | 5’CTTTCGTGCAGGCCTTTG3’ | 5’TGTAGCTGCCTCGTGCTG3’ |
| *Mme* | 5’CCCAGGAGTTCTGTTTGGTG3’ | 5’CTTTCTGATCTTCCCATCACCT3’ |
| *Hnmt* | 5’TGCCAATGCTAAAATTCTCATTAT3’ | 5’TGAGCAAGATCAGAAGATGTGAC3’ |
| *Tnfrsf1b* | 5’AAGGGTCTCAGGAAGCCTGT3’ | 5’GGCTTGGGAAGAACACTGAG3’ |
| *Trpa1* | 5’TGGAAATACTGAAACAGAAATACCG3’ | 5’CTGTCTTGGAAAGAGCAATGG3’ |
